# Recombinant Human Prion Protein Inhibits Prion Propagation *in vitro*

**DOI:** 10.1038/srep02911

**Published:** 2013-10-09

**Authors:** Jue Yuan, Yi-An Zhan, Romany Abskharon, Xiangzhu Xiao, Manuel Camacho Martinez, Xiaochen Zhou, Geoff Kneale, Jacqueline Mikol, Sylvain Lehmann, Witold K. Surewicz, Joaquín Castilla, Jan Steyaert, Shulin Zhang, Qingzhong Kong, Robert B. Petersen, Alexandre Wohlkonig, Wen-Quan Zou

**Affiliations:** 1 Department of Pathology, Case Western Reserve University School of Medicine, Cleveland, Ohio, USA; 2 Department of Neurology, Case Western Reserve University School of Medicine, Cleveland, Ohio, USA; 3 National Prion Disease Pathology Surveillance Center, Case Western Reserve University School of Medicine, Cleveland, Ohio, USA; 4 National Center for Regenerative Medicine, Case Western Reserve University School of Medicine, Cleveland, Ohio, USA; 5 VIB, Department of Structural Biology, Vrije Universiteit Brussels, Belgium; 6 Structural Biology Brussels, Vrije Universiteit Brussels, Belgium; 7 The First Affiliated Hospital, Nanchang University, Nanchang, Jiangxi Province, The People's Republic of China; 8 Biophysics Laboratories, Institute of Biomedical and Biomolecular Sciences, University of Portsmouth, Portsmouth, United Kingdom; 9 Hôpital Lariboisière, Service d'Anatomie et Cytologie Pathologiques, Paris Denis Diderot University, Paris, France; 10 IRB - Hôpital ST ELOI, CHU de Montpellier, Montpellier, France; 11 Department of Neuroscience, Case Western Reserve University School of Medicine, Cleveland, Ohio, USA; 12 CIC bioGUNE and IKERBASQUE, Basque Foundation for Science, 48160 Derio and 48011 Bilbao, Bizkaia, Spain; 13 Department of Physiology and Biophysics, Case Western Reserve University School of Medicine, Cleveland, Ohio, USA; 14 National Institute of Oceanography and Fisheries (NIFO), Cairo, Egypt; 15 These authors contributed equally to this work.

## Abstract

Prion diseases are associated with the conformational conversion of the cellular prion protein (PrP^C^) into the pathological scrapie isoform (PrP^Sc^) in the brain. Both the *in vivo* and *in vitro* conversion of PrP^C^ into PrP^Sc^ is significantly inhibited by differences in amino acid sequence between the two molecules. Using protein misfolding cyclic amplification (PMCA), we now report that the recombinant full-length human PrP (rHuPrP23-231) (that is unglycosylated and lacks the glycophosphatidylinositol anchor) is a strong inhibitor of human prion propagation. Furthermore, rHuPrP23-231 also inhibits mouse prion propagation in a scrapie-infected mouse cell line. Notably, it binds to PrP^Sc^, but not PrP^C^, suggesting that the inhibitory effect of recombinant PrP results from blocking the interaction of brain PrP^C^ with PrP^Sc^. Our findings suggest a new avenue for treating prion diseases, in which a patient's own unglycosylated and anchorless PrP is used to inhibit PrP^Sc^ propagation without inducing immune response side effects.

Prions are infectious pathogens that cause a group of transmissible prion diseases in animals and humans. Currently there is no cure for prion diseases, largely because the molecular mechanism underlying prion formation is poorly understood. The scrapie isoform (PrP^Sc^) of the cellular prion protein (PrP^C^) is the only known component of prions. The conversion of PrP^C^ into PrP^Sc^ constitutes a key molecular event in the pathogenesis of prion diseases; however, the mechanism underlying the conversion remains unclear. It has been proposed that prion formation occurs in a template-assisted process involving the physical interaction of the PrP^Sc^ template and the PrP^C^ substrate^1^. Indeed, early *in vivo* studies indicated that interaction between non-homologous PrP molecules inhibits the disease process^2^,^3^,^4^. The incorporation of chimeric PrP into PrP^Sc^ was influenced by the PrP sequence in scrapie-infected cell lines expressing chimeric mouse-hamster PrP^5^. Subsequently, Priola et al provided direct evidence that heterologous PrP molecules, which differed by as little as one residue, interfere with the generation of PrP^Sc^ in scrapie-infected mouse cells (ScN2a)^6^. Based on this result, as well as previous studies, the authors proposed three possible mechanims for the interference. First, interaction between dissimilar PrP^Sc^ and PrP^C^ molecules might slow the aggregation and accumulation of PrP^Sc^ by interfering with the interaction of similar PrP monomers^7^,^8^,^2^. Second, incorporation of non-homologous PrP molecules into PrP^Sc^ aggregates might lead to a destabilization of the aggregates^9^. Finally, exogenous PrP molecules might inhibit the interaction of the endogenous PrP with cellular ligands^10^.

Studies with transgenic mice expressing human or mouse/human chimeric PrP implied that a species-specific cofactor, termed protein X, is necessary for PrP^Sc^ formation^11^. Four mouse specific substitutions in the C-terminal region of PrP, including residues 167, 171, 214, and 218 were identified that inhibit the conversion of wild-type PrP^C^ in a dominant-negative manner in scrapie-infected cells^12^. These residues were proposed to form a discontinuous epitope that interacts with protein X. However, although many putative protein X genes have been proposed, knockout of these genes in mice failed to significantly alter incubation times^13^. Moreover, the recombinant Q218K variant, one of the four dominant negative mutants, inhibited the polymerization of recombinant wild-type PrP in the absence of protein X^14^. The dominant-negative effect observed in pure recombinant molecules was presumably mediated by physical interaction between the Q218K variant and wild-type PrP. Using the protein misfolding cyclic amplification (PMCA) assay with wild-type and mutant PrP expressed in Chinese hamster ovary cells as substrates, Geoghegan et al further demonstrated that trans-dominant inhibition of prion propagation *in vitro* was not mediated by an accessory cofactor and proposed that PrP molecules compete for binding to a nascent seeding site on newly formed PrP^Sc^ molecules^15^.

In the current study, we demonstrate that unglycosylated and anchorless recombinant full-length human PrP23-231 is able to dramatically inhibit human PrP^Sc^ amplification *in vitro*. Moreover, this inhibition also occurs in a scrapie-infected cell model. Although to a lesser extent, recombinant PrP from other species also inhibits human PrP^Sc^ amplification. We show that the inhibition may depend on direct interaction of the inhibitory recombinant PrP with human PrP^Sc^ using a capture strategy.

## Results

### Amplification of human PrP^Sc^ is inhibited by unglycosylated and anchorless recombinant human PrP

**Figure 1:**
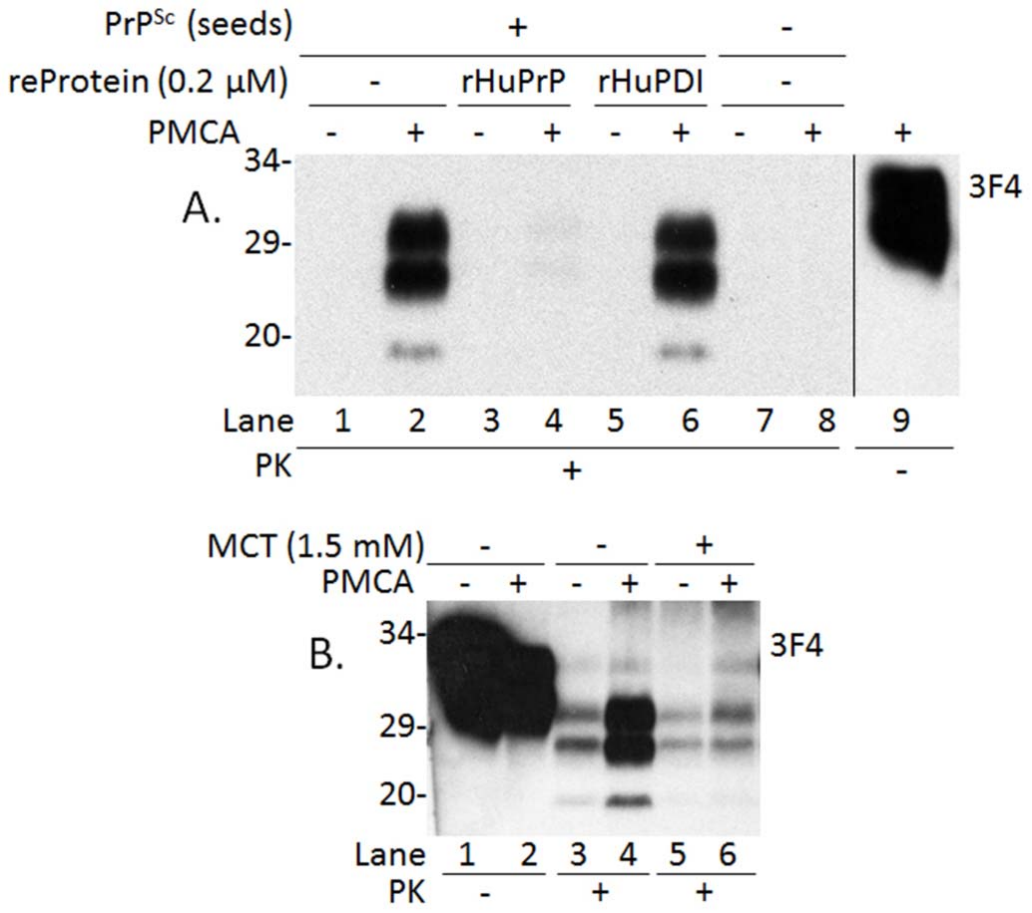
Inhibition of amplification of human PrP^Sc^ by recombinant human PrP (rHuPrP23-231) and mechlorethamine (MCT). (A) Amplification of human PrP^Sc^ from iatrogenic CJD carrying valine (V)/valine polymorphism at residue 129 (129VV) of PrP characteristic of PrP^Sc^ type 2 (iCJDVV2, seeds) was carried out by PMCA in the presence of uninfected brain homogenates from humanized transgenic mice expressing PrP-129V (substrates). Lanes 1 and 2: Positive PMCA control without inhibitors; Lanes 3 and 4: PMCA in the presence of 0.2 μM of rHuPrP23-231 with methionine at polymorphic residue 129 (129M); Lanes 5 and 6: PMCA in the presence of 0.2 μM of recombinant human protein disulfide isomerase (rHuPDI); Lanes 1 through 6: PMCA with PrP^Sc^ seeds; Lanes 7 and 8: PMCA without PrP^Sc^ seeds and inhibitors; Lane 9: The PrP sample without PK-treatment; and Lanes 1 through 8 with PK-treatment. Samples without (−) or with (+) PMCA were treated with 100 μg/ml PK prior to SDS-PAGE and Western blotting with 3F4. Intense PrP^Sc^ is detectable in lane 2 (positive control) but not in lane 8 (negative control). However, amplification is virtually undetectable in the presence of rHuPrP23-231 (lane 4) while detectable in the presence of rHuPDI (lane 6). The blot is a representative of five independent experiments. (B) Amplification of human PrP^Sc^ from iCJD was carried out in the absence (lanes 3 and 4) or presence (lanes 5 and 6) of MCT. Amplification of PrP^Sc^ is inhibited in the presence of MCT (1.5 mM) compared to the sample in the absence of MCT. Lanes 3 through 6 were treated with PK while lanes 1 and 2 were not. The blot is a representative of three independent experiments.

A recent study in our lab suggested that the glycoform-selective prion formation observed in unique sporadic and familial forms of prion disease may involve changes in N-linked glycosylation^16^. Indeed, using the serial PMCA, Nishina et al. observed that the formation of Sc237 hamster prions was dependent on substoichiometric levels of unglycosylated PrP^C^ molecules isolated from the hamster brain^17^. Moreover, recombinant hamster PrP that lacks both glycans as well as the glycophosphatidylinositol (GPI) anchor was found to inhibit amplification of hamster PrP^Sc^^18^,^19^ in a standard PMCA reaction. To investigate the effect of unglycosylated and anchorless PrP on human PrP^Sc^ formation, we performed the PMCA assay in which human PrP^Sc^ from brain homogenates of an iatrogenic CJD (iCJD) was used as the seed while human PrP^C^ from brain homogenates of transgenic mice expressing wild-type human PrP-129V was used as the substrate. The unglycosylated and anchorless recombinant full-length human PrP23-231 (rHuPrP23-231) with methionine at the polymorphic residue 129 was added into the PMCA. In controls lacking rHuPrP23-231, intense PK-resistant PrP^Sc^ (PrP^res^) bands were detected in the sample subjected to PMCA while virtually no PrP was detectable in the non-PMCA sample, suggesting that significant amplification of PrP^Sc^ occurred (Figure 1A, lanes 1 and 2). In contrast, in the sample that contained no PrP^Sc^ seeds, PK-resistant PrP (PrP^res^) was not detectable (Figure 1A, lanes 7 and 8). In the presence of 0.2 μM rHuPrP23-231, virtually no PrP^res^ bands were detectable in the sample subjected to PMCA, similar to the non-PMCA sample, indicating that rHuPrP23-231 inhibited the amplification of PrP^Sc^. In order to assess whether other proteins known to interact with PrP could inhibit the amplification of PrP^Sc^, we used human protein disulfide isomerase (PDI)^20^. When PMCA was performed in the presence of the same amount of recombinant PDI (rPDI), intense PrP^res^ bands were still detected, similar to the sample without any recombinant proteins. Thus, the inhibition is rHuPrP-specific. To investigate whether the methionine (M) or valine (V) polymorphism at residue 129 affects the inhibition efficiency, we replaced the 129M-rHuPrP with an equal amount of 129V-rHuPrP in the PMCA reaction. No significant difference in the inhibition efficiency was observed (data not shown). As a result, in subsequent experiments we continued using 129M-rHuPrP. Our previous study found that an anti-tumor drug mechlorethamine (MCT) inhibited hamster PrP^Sc^ 263K amplification using PMCA^21^. Here we determined the effect of MCT on human PrP^Sc^ amplification by using the same substrates and seeds in the absence or presence of MCT. The intensity of PK-resistant PrP was significantly decreased in the sample containing MCT compared to the sample without MCT (Figure 1B). Thus, we confirmed that MCT also inhibits amplification of human PrP^Sc^ (Figure 1B).

### The effect of rHuPrP23-231 on PrP^Sc^ amplification is dose-dependent

**Figure 2:**
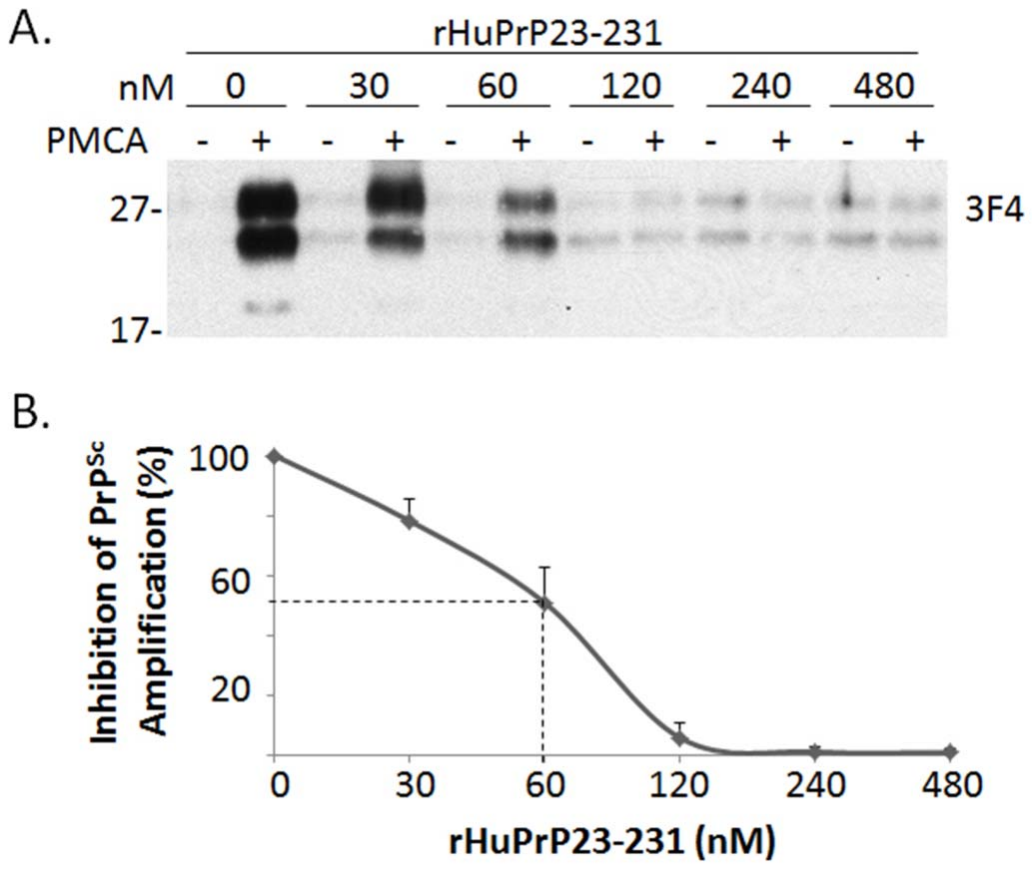
Dose-dependent inhibition of PrP^Sc^ amplification by rHuPrP23-231. (A) PMCA was performed with the mixture of human PrP^Sc^ (seeds) from iCJDVV2 and brain homogenates from TgWV (substrates) in the presence of different amounts of rHuPrP23-231 ranging from 0 to 480 nM. Samples without (−) or with (+) PMCA were subjected to PK-treatment prior to Western blotting with 3F4. (B) Percentage of inhibition of PrP^Sc^ amplification is a function of concentrations of rHuPrP23-231 added. The inhibition of PrP^Sc^ amplification by recombinant rHuPrP23-231 is dose-dependent and the half maximal effective concentration (EC50) is approximately 60 nM. The results are a representative of three independent experiments.

To determine whether there is a dose-dependent effect on PrP^Sc^ amplification, we next conducted PMCA in the presence of different concentrations of rHuPrP23-231. Different amounts of rHuPrP23-231 ranging from 0 to 480 nM were added to the reaction containing both PrP^Sc^ seeds and PrP^C^ substrates immediately before PMCA. The intensity of PrP^res^ detected in samples subjected to PMCA was decreased as a function of the concentration of rHuPrP23-231 added (Figure 2A). The half maximal effective concentration (EC50) of the inhibition of PrP^Sc^ amplification by rHuPrP23-231 was approximately 60 nM (Figure 2B).

### Inhibition of PrP^Sc^ amplification by rHuPrP23-231 is species-specific

**Figure 3:**
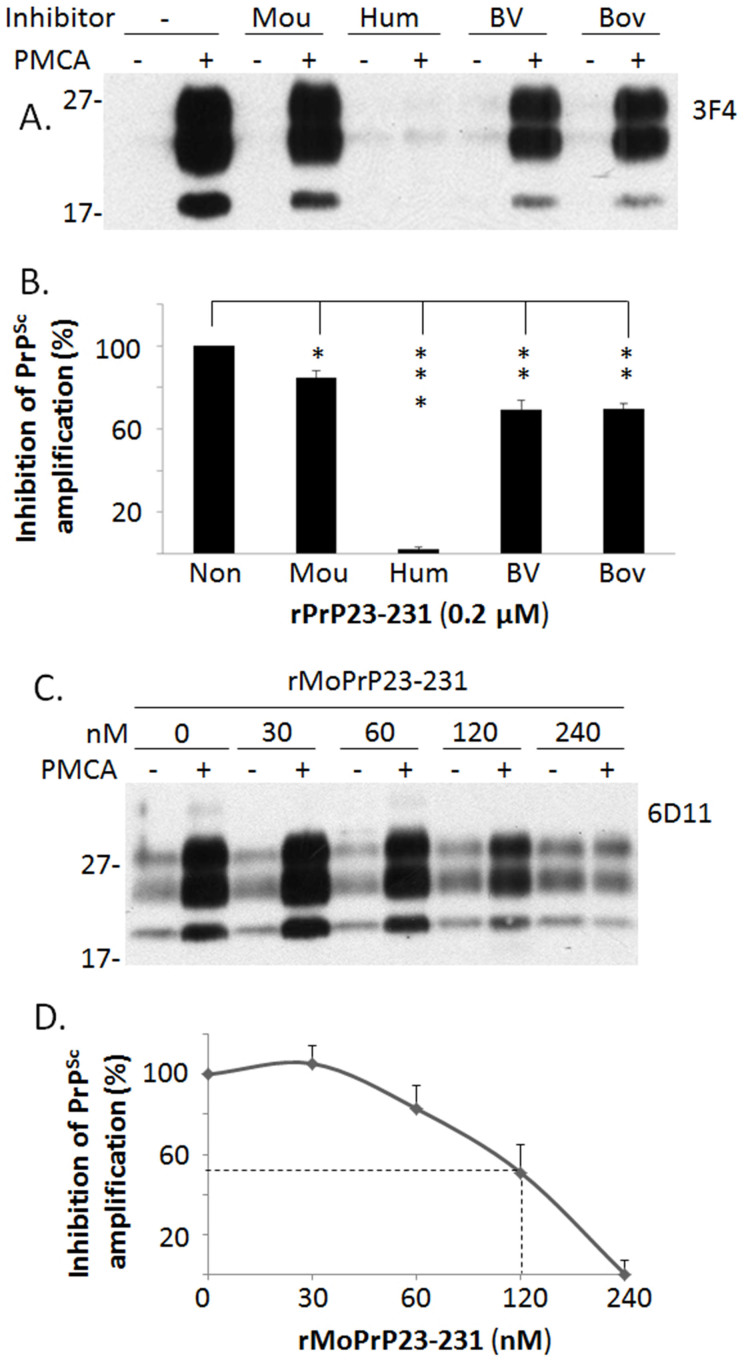
Inhibition of PrP^Sc^ amplification by different species of recombinant PrP. (A) PMCA was performed with the mixture of human PrP^Sc^ (seeds) from iCJDVV2 and brain homogenates from TgWV (substrates) in the presence of different species of recombinant PrP (0.2 μM each) including mouse (Mou: rMoPrP23-231 with 129M, home-made), human (Hum: rHuPrP23-231 with 129M), bank vole (BV: rBvPrP23-231 with 109I), and bovine (Bov: rBoPrP23-231 with 129M). (B) Inhibition of PrP^Sc^ amplification was quantified using densitometric analysis based on three independent experiments. Bars represent the percentage of all five amplified PrP^Sc^ with or without inhibitors to the amplified PrP^Sc^ without any inhibitors. Amplified PrP^Sc^ was calculated by subtracting the untreated PrP intensity (−) from the PMCA-treated PrP intensity (+) shown in (A). Of all recombinant PrP species examined, recombinant human PrP23-231 exhibited the highest inhibition compared to other species (**: *p* < 0.01; ***: *p* < 0.001). (C) PMCA was performed with mouse brain homogenates infected with prion 139A (seeds) and brain homogenates from wild-type mouse FVB (substrates) in the presence of different concentrations of the commercially-derived rMoPrP23-231 with 129M. (D) The inhibition of mouse prion 139A is dose-dependent and the half maximal effective concentration (EC50) is approximately 120 nM, which is based on three independent experiments.

We then determined whether recombinant PrP from other species also inhibits human PrP^Sc^ amplification. The same concentration of recombinant human, mouse, bank vole, and bovine PrP species was used. Compared to the PMCA sample in which no recombinant PrP was added, the samples that contained different recombinant PrP species exhibited varying degrees of inhibition. The efficiency of inhibition by the heterologous recombinant PrP species was much lower than that of the homologous rHuPrP23-231 (~20–30% vs ~100%) (Figure 3A and 3B). Therefore, the inhibition of PrP^Sc^ amplification by rHuPrP23-231 was species-specific. Indeed, although 200 nM recombinant mouse PrP23-231 (rMoPrP23-231) resulted in approximately 15% inhibition of human PrP^Sc^ amplification (Figure 3A and 3B), the same amount of rMoPrP23-231 inhibited more than 50% of mouse PrP^Sc^ amplification (Figure 3C and 3D). The EC50 of rMoPrP23-231 in the inhibition of mouse PrP^Sc^ (mouse 139A prion strain) was approximately 120 nM (Figure 3D). However, rHuPrP23-231 did not significantly inhibit amplification of mouse PrP^Sc^ in a standard PMCA reaction (Figure S1).

### Effect of truncated PrP and PrP^C^- or PrP^Sc^-specific binding reagents on human PrP^Sc^ amplification

**Figure 4:**
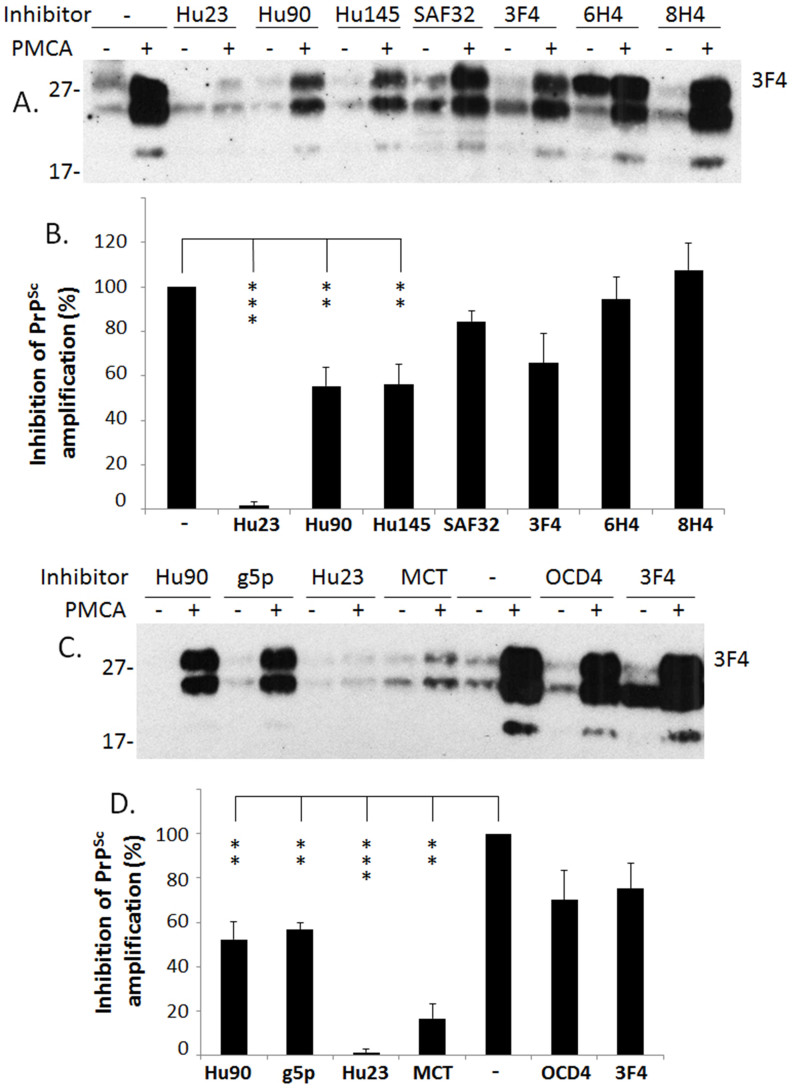
Inhibition of human PrP^Sc^ amplification by truncated recombinant human PrP and different anti-PrP antibodies. (A) PMCA was performed with the mixture of human PrP^Sc^ (seeds) from iCJDVV2 and brain homogenates from TgWV (substrates) in the presence of rHuPrP23-231 (Hu23), rHuPrP90-231 (Hu90), rHuPrP23-145 (Hu145), or different antibodies against PrP (0.1 μM each), respectively. The four antibodies include SAF32 against human PrP59-89, 3F4 against PrP105-112, 6H4 against PrP145-152, and 8H4 against PrP175-185. The result is a representative of three independent experiments. (B) Inhibition of PrP^Sc^ amplification was quantified using densitometric analysis based on three independent experiments. Of all recombinant PrP species and anti-PrP antibodies examined, recombinant human PrP23-231 exhibited the highest inhibition compared to others (**: *p* < 0.01; ***: *p* < 0.001). (C) PMCA was performed with the mixture of human PrP^Sc^ (seeds) from iCJDVV2 and brain homogenates from TgWV (substrates) in the presence of Hu23, Hu90, g5p, MCT, OCD4, or 3F4 (0.1 μM each), respectively. The Western blot shown is a representative of three independent experiments. (D) Inhibition of PrP^Sc^ amplification was quantified using densitometric analysis based on three independent experiments. In addition to rHuPrP23-231 and rHuPrP90-231, g5p and MCT also significantly inhibited PrP^Sc^ amplification (**: *p* < 0.01; ***: *p* < 0.001), whereas OCD4 and 3F4 did not (*p* > 0.05).

We further determined which part of recombinant PrP is involved in the inhibition and investigated the effect of PrP^C^- or PrP^Sc^-binding reagents on human PrP^Sc^ amplification. This included N-terminally-truncated recombinant human PrP90-231(Hu90), C-terminally-truncated recombinant human PrP23-145 (Hu145), and anti-PrP antibodies such as SAF32, 3F4, 6H4, and 8H4. We also investigated the effect of an anti-DNA antibody OCD4 and the gene 5 protein (g5p, a single stranded DNA-binding protein) that were previously shown to specifically bind to PrP^Sc^ but not to PrP^C^^22^. Compared to the PrP^res^ intensity from the sample in the absence of recombinant PrP or antibodies, the PrP^res^ intensity was decreased approximately 50% or more when rHuPrP90-231, rHuPrP23-145, g5p, or MCT were added to the reaction (*p* < 0.01 for Hu90, Hu145, or MCT; *p* < 0.001 for Hu23) (Figure 4A through 4D). PrP^res^ was decreased approximately 10%–30% when SAF32, 3F4, 6H4, or OCD4 was added to the reaction, which was not statistically significant compared to the control containing no antibody (*p* > 0.05). A slight increase in the level of PrP^res^ (~5%–10%) was observed in the sample containing the 8H4 antibody (*p* > 0.05). These results suggest that the inhibition of PrP^Sc^ amplification involves both N- and C-terminal domains of the protein. The SAF32, 3F4 and 6H4 antibodies that exhibited some inhibition are against residues 78–91, 106–112, and 145–152, respectively, while the 8H4 antibody that exhibited no inhibition is against residues 175–185 in human PrP. Again, rHuPrP23-231 (Hu23) virtually completely inhibited PrP^Sc^ amplification. It is most likely that the inhibitory sites are N-terminal to residue 175. Moreover, although both g5p and OCD4 specifically captured PrP^Sc^, the efficiency of PrP^Sc^ inhibition was greater with g5p than with OCD4, suggesting that the two may have different binding sites on the PrP^Sc^ molecule. A second source of rHuPrP23-231 and rHuPrP90-231 generated previously^23^ was also examined and there were no significant differences in the inhibitory efficiency between currently and previously generated recombinant proteins (data not shown).

### Recombinant rHuPrP23-231 also inhibits murine PrP^Sc^ propagation in scrapie-infected mouse neuroblastoma cells (ScN2a)

**Figure 5:**
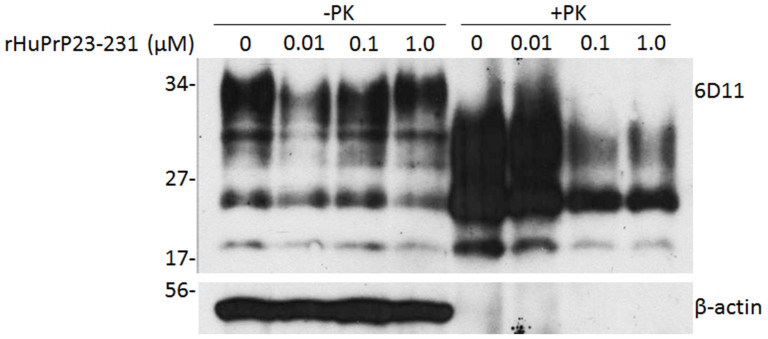
Inhibition of PrP^Sc^ propagation by recombinant human PrP23-231 in ScN2a cells. Different amounts of rHuPrP23-231 ranging from 0 to 1 μM were added into the cell culture medium for four days. The cells were lysed and subjected to PK-digestion at 25 μg/ml prior to SDS-PAGE and Western blotting with 6D11. The intensity of PK-resistant PrP^Sc^ was significantly decreased at 0.1 μM of rHuPrP23-231 or greater. β-actin was determined to normalize the levels of individual samples examined. The blot is a representative of three independent experiments.

We next determined whether rHuPrP23-231 is able to inhibit PrP^Sc^ propagation in scrapie-infected cells. Mouse prion-infected ScN2a cells were chosen in our study, due to the lack of human cell models of prion infection. This cell line is the most widely-used cell model, not only for studying the cell biology of prion replication, but also for screening therapeutic compounds^24^,^25^. ScN2a cells were incubated with concentrations of rHuPrP23-231 ranging from 0 to 1 μM for four days. While the cell toxicity, conducted as previously described^26^, was not observed in the cells incubated with rHuPrP23-231 at these concentrations, PrP^res^ was decreased as a function of the increased concentration of recombinant PrP added to the media (Figure 5). No significant changes in the levels of β-actin were observed; β-actin was used to normalize the protein loading of the cell lysates.

### Recombinant HuPrP23-231 binds to brain PrP^Sc^ but not PrP^C^

**Figure 6:**
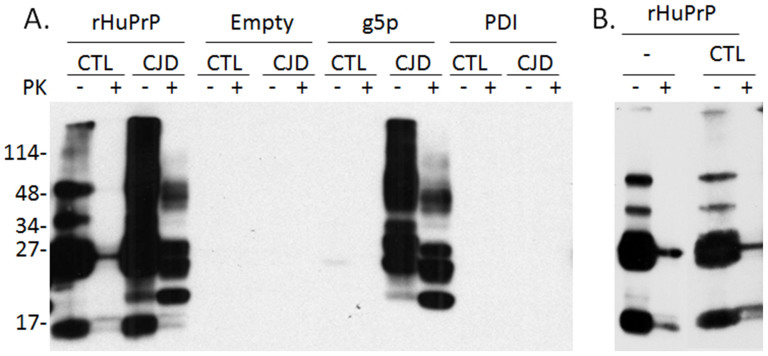
Binding of recombinant human PrP23-231 to human PrP^Sc^. (A) Capture of PrP^Sc^ or PrP^C^ by rHuPrP23-231 was performed by incubation of rHuPrP23-231-conjugated magnetic beads with uninfected (CTL) and iCJD human brain homogenates, respectively. Magnetic beads without conjugated proteins were used as negative control (Empty). The g5p-conjugated beads were used as a positive control (g5p). To determine the specificity of the binding, we also examined the beads conjugated with recombinant PDI. The PK-resistant PrP^Sc^ was only detected in the preparations captured by rHuPrP23-231 and g5p beads from CJD brain homogenates. In contrast, no PK-resistant PrP was detected in CJD samples captured by empty beads and PDI beads. No PrP signal was detected in the uninfected samples captured by all the beads except the rHuPrP beads. The bands detected in the preparation captured by rHuPrP beads are expected to be the recombinant PrP itself. (B) To determine whether PrP detected in the preparation captured by rHuPrP from the uninfected brain homogenate shown in panel (A) was brain PrP^C^ or rHuPrP itself, the capture experiment was also performed in the binding buffer alone without uninfected brain homogenate. The virtual same PrP bands were detected in both capture experiments in the absence and presence of uninfected human brain homogenates, suggesting that the detected PrP bands were from rHuPrP itself and no brain PrP^C^ was captured. The blots were probed with 3F4. The results shown in (A) and (B) are a representative of two experiments.

To determine whether the inhibition of PrP^Sc^ replication by rHuPrP23-231 results from its interaction with PrP^C^, PrP^Sc^ and/or both, we used a magnetic bead-based capture assay as previously described^27^. g5p- or PDI-beads were used as controls. No PrP was detected in the sample from the normal brain control after PK-treatment, although several bands were detected using the 3F4 antibody (Figure 6A). In contrast, a smear above 27 kDa was detected in the sample from sCJD; moreover, the three typical PrP^res^ bands were detectable after PK-treatment (Figure 6A). Beads without any conjugated protein or antibody (empty) or conjugated with PDI did not capture PrP from either uninfected or prion-infected brain homogenates. As expected^22^,^27^, g5p-conjugated beads captured PrP^Sc^ (Figure 6A). To confirm that rHuPrP23-231 cannot capture PrP^C^, we incubated rHuPrP23-231-conjugated beads with normal brain homogenate or binding buffer alone. No differences were found between the two conditions (Figure 6B). The four major bands were monomeric or dimeric recombinant full-length or truncated PrP species, respectively. Therefore, our results indicate that recombinant PrP23-231, just like g5p, binds specifically to PrP^Sc^, but not to PrP^C^.

## Discussion

The *in vitro* and *in vivo* conversion efficiency of PrP^C^ into PrP^Sc^ can be significantly affected by the presence of additional PrP molecules that differ from the endogenous PrP^C^ by as little as one residue^6^,^28^,^11^,^12^. The present study now demonstrates that a PrP molecule that shares the identical amino acid sequence with the PrP^C^ substrate and PrP^Sc^ template also causes interference. It is worth noting that although the amino acid sequence is identical, recombinant PrP does not contain N-linked glycans or a GPI anchor. Our new results suggest that in addition to the amino acid sequence, glycosylation and the GPI anchor are important in mediating the conversion of PrP^C^ into PrP^Sc^. The unglycosylated and anchorless recombinant PrP appears to act as an inhibitor of the conversion process by preferentially binding to PrP^Sc^.

PrP^C^ is a glycoprotein with two non-obligatory, N-linked glycosylation sites at residues 181 and 197 and a GPI anchor^24^,^29^,^30^. Binding of heterologous PrP^C^ to PrP^Sc^ can be influenced by PrP^C^ glycosylation in a species-specific manner^31^,^32^. Moreover, using PMCA and a scrapie cell assay, Nishina et al reported that the stoichiometry of host PrP^C^ glycoforms modulates the efficiency of PrP^Sc^ formation *in vitro*^17^. Specifically, their study demonstrated that while unglycosylated PrP^C^ is required to propagate mouse RML prions, in a similar reaction, amplification of hamster Sc237 prions is inhibited by substoichiometric levels of homologous unglycosylated PrP^C^. This study provides direct *in vitro* evidence that changes in the PrP glycoform ratios can affect the efficiency of PrP^Sc^ formation in a species-specific manner. Recently, we observed glycoform-selective prion formation in unique sporadic and inherited forms of Creutzfeldt-Jakob disease (CJD) including variably protease-sensitive prionopathy (VPSPr) and familial CJD linked to a valine to isoleucine mutation at residue 180 (fCJD^V180I^)^16^. Although all four glycoforms are present, including di-, monoglycosylated at residue 181 (mono-181), monoglycosylated at residue 197 (mono-197), and unglycosylated PrP forms in the brain of VPSPr and fCJD^V180I^, only the mono-197 and unglycosylated PrP species were converted into PrP^Sc^. The mono-181 and diglycosylated PrP species were not converted into PrP^Sc^ in the cerebral cortical brain areas examined. Moreover, the level of the classic PK-resistant PrP^Sc^ probed with the 3F4 antibody was significantly decreased compared to typical sporadic CJD. Instead, a unique five-step ladder-like electrophoretic profile of PK-resistant PrP^Sc^ was detected in both diseases by the 1E4 antibody^16^. In contrast to the threonine to alanine mutation at residue 183 of PrP (PrP^T183A^), the PrP^V180I^ mutation exhibits a typical PrP glycosylation profile, although there is no detectable mono-181 and diglycosylated PrP^Sc^^33^,^16^. However, using the N-linked glycosylation prediction algorithm NetNGlyc 1.0 at http://www.cbs.dtu.dk/services/NetNGlyc/34, we predicted a slight decrease in the glycosylation potential at N181 in PrP^V180I^ compared to PrP^Wt^ (0.597 vs 0.664) while no potential change was predicted at all for N181 in PrP^T183A^^16^. The prediction data suggests that although the T183A mutation completely eliminates the N181 glycosylation site, the V180I mutation may merely alter the glycan composition at N181, which modifies the ratio of the four PrP glycoforms in the PrP mixture. Further investigation into the mechanism, by which altered glycosylation affects both conversion efficiency of PrP^C^ into PrP^Sc^ and PrP^Sc^ conformation, is warranted. Unglycosylated and anchorless recombinant human PrP may have greater affinity for PrP^Sc^ seeds compared to the brain PrP^C^, but it is a poor substrate for conversion into PrP^res^ by the standard PMCA protocol or in ScN2a cells. Indeed, both recombinant hamster and mouse PrP are not converted into PK-resistant PrP by serial PMCA in the presence of hamster prion Sc237 or mouse prion RML, respectively^17^. It is worth noting, however, that the recombinant PrP could be converted into PrP^res^ using a modified PMCA protocol in which the conversion buffer contained 0.1% SDS and the normal brain-derived PrP^C^ was replaced by recombinant hamster PrP as a substrate^35^,^36^ and the product of this reaction was proved to be infectious in animal bioassays^37^. Furthermore, in the absence of brain homogenates, recombinant PrP was converted by PMCA to highly infectious prions in the presence of additional cofactors such as phosphatidylglycerol and RNA^38^ or phosphatidylethanolamine^39^. Prion infectivity was also produced in Syrian hamsters by inoculating full-length recombinant hamster PrP that was converted into a cross-β-sheet amyloid conformation and subjected to an annealing procedure^40^.

We cannot rule out the possibility that the inhibition of human PrP^Sc^ amplification by recombinant human PrP results from the lack of the GPI anchor, although the GPI anchor of PrP^C^ is believed to have little or no effect on the formation of PK-resistant PrP^31^,^32^. Anchorless PrP generated in either cultured mammalian cells or E. coli are converted to PK-resistant PrP by a cell-free conversion approach^41^,^42^,^43^,^44^. Moreover, it has been reported that anchorless prion protein induced an infectious amyloid disease in transgenic animals, although the animal themselves were asymptomatic^45^. However, amplification of hamster PrP^Sc^ using a standard PMCA protocol is inhibited when the substrate of normal hamster brain PrP^C^ was pretreated with phosphatidylinositol-specific phospholipase C (PIPLC) to remove the GPI anchor^19^. Furthermore, recombinant hamster PrP was previously shown to inhibit PMCA of hamster PrP^Sc^ using the normal hamster brain homogenate as a substrate^18^. Kim et al proposed that both of these effects are due to the lack of the GPI anchor in PIPLC-treated PrP^C^ or recombinant PrP^19^. On the other hand, the unglycosylated hamster PrP^C^ purified from brains and containing an intact GPI anchor likewise inhibits amplification of Sc237 prions^17^. Therefore, the role of GPI anchor in the inhibition of human and mouse PrP^Sc^ propagation by recombinant human PrP observed in our study remains to be determined.

We demonstrated that recombinant human and other PrP that exhibited 50% or greater inhibition of PrP^Sc^ formation in a PMCA reaction bind to human PrP^Sc^ but not to PrP^C^. Although full-length, N- or C-terminally truncated recombinant PrP all bind to PrP^Sc^ efficiently, the full-length rHuPrP23-231 exhibits the highest inhibition efficiency compared to the two truncated forms, suggesting that the inhibition involves both N- and C-terminal domains. Moreover, antibodies including SAF32, 3F4, and 6H4 directed against PrP regions covering residues 59 to 152 showed less than 10% inhibition. The 8H4 antibody against human PrP175-185 exhibited virtually no inhibition. These results are in good agreement with a previous report by Horiuchi and Caughey^1^. In addition, it has been shown that the 3F4 and 6H4 antibodies preferentially bind to native PrP^C^, although they also detect denatured PrP^Sc^ on Western blots^46^,^47^. Therefore, the interaction of inhibitors with PrP^Sc^ may be required for the inhibition of PrP^C^ conversion. We observed that recombinant mouse PrP is also able to bind to human PrP^Sc^ (Figure S2), although it caused significantly less inhibition compared to recombinant human PrP. Moreover, the anti-DNA antibody that specifically captures PrP^Sc^ but not PrP^C^ showed less than 10% inhibition while g5p caused more than 50% inhibition. This suggests that the different inhibitors have distinct binding sites on PrP^Sc^: one class of sites is specifically associated with recruiting PrP^C^ while the other is not. Recombinant human PrP is likely to compete with brain PrP^C^ for the same site on the PrP^Sc^ molecule. Moreover, its affinity for PrP^Sc^ seems to be greater than that of brain-derived PrP^C^. Interestingly, a two-site model has been proposed by Horiuchi and co-workers to explain the molecular mechanism for sequence-difference interference^28^. According to this model, PrP^Sc^ has two types of PrP^C^ binding sites: one is able to induce conversion to PrP^Sc^ while the other is not.

A recombinant mouse PrP with a substitution of lysine for glutamine at mouse codon 218 (rPrP-Q218K), corresponding to human PrP^E219K^, an Asian-specific polymorphism believed to be resistant to CJD infection, considerably prolonged incubation time of prion infection in an iatrogenic mouse model^48^. Recombinant mouse PrP was delivered into the mouse brain for 7 days by intracerebroventricular administration using an indwelling catheter connected to an implanted osmotic pump. The same group also found that rPrP-Q218K reduced PrP^Sc^ formation in ScN2a cells. However, using wild-type mouse PrP did not cause inhibition, which is different than our findings. This discrepancy may be due to different experimental conditions between the studies. We showed that murine PrP^Sc^ amplification was inhibited in both PMCA and ScN2a by unglycosylated and anchorless recombinant human PrP. Most importantly, since the amino acid sequence of recombinant human PrP is identical to that of human brain PrP^C^, it is expected that this protein would not elicit an immune response after intracerebroventricular administration while it inhibits PrP^Sc^ propagation. Therefore, our findings suggest a new therapeutic strategy for treating human prion diseases.

## Methods

### Recombinant prion protein, protein disulfide isomerase and mechlorethamine

The various constructs for producing recombinant protein: human PrP [rHuPrP23-231 or rHuPrP90-231 with methionine at polymorphic residue 129 (129M)], mouse PrP (rMoPrP23-231), or bank vole PrP [rBvPrP23-231 with isoleucine at polymorphic residue (109I)] were cloned into the pET28a vector (Merck Millipore) and expressed and purified as a soluble protein as previously described^49^. Another set of recombinant human PrPs including rHuPrP23-231 and rHuPrP90-231 with 129M or 129V described previously^23^ was used to confirm the results with recombinant PrP generated in this study. The protein disulfide isomerase (PDI) plasmid was a generous gift from Dr. Joris Messens. Expression and purification of PDI were performed as previously described^50^. rHuPrP (23-145) was kindly provided by Dr. Giuseppe Legname. Recombinant mouse (rMoPrP23-231, the second source) and bovine PrP (rBoPrP23-231) were purchased from Prionics AG (Zurich, Switzerland). Mechlorethamine (MCT) was purchased from Sigma-Aldrich (Milwaukee, WI, USA).

### Construction of transgenes expressing human PrP-129V

The transgene constructs were based on the murine half-genomic PrP clone in plasmid pHGPRP^51^. The HuPrP-129V open reading frame (ORF) was amplified from the human genomic DNAPAC (P1-derived artificial chromosome) clone RP5–1068H6 (obtained from the Sanger Center, Cambridge, UK) with primers HRM-F (TATGTGGACTGATGTCGGCCTCTGCAAGAAGCGC) and HRM-R (CCACCTCAATTGAAAGGGCTGCAGGTGGATAC). The PCR product was digested with *Psh*AI and *Mfe*I and used to replace the corresponding 0.97 kb *Psh*AI–*Mfe*I fragment in pHGPRP to create pHGHuPrP-129V. The inserted 0.97 kb *Psh*AI–*Mfe*I fragment in pHGHuPrP-129V was then sequenced with the primers HRM-R, HRM-F, and HP306R (CATGTTGGTTTTTGGCTTACTC). One error free clone was chosen for the creation of transgenic mice.

### Generation, screening, and characterization of transgenic Tg(HuPrP-129V)Prnp^0/0^ mice (TgWV)

The 12.2 kb HuPrP-129V transgene construct was microinjected into fertilized FVB/NJ eggs, and planted into the oviducts of pseudopregnant CD-1 mice at the transgenic mouse facility of Case Western Reserve University (Cleveland, OH). Founder pups were screened by PCR of tail DNA. All founder mice carrying the transgene were bred with FVB/*Prnp^0/0^* mice^51^ to obtain Tg mice in PrP-null background. Transgenic PrP expression in the brain and other tissues of the Tg mice was examined by Western blot analysis using monoclonal antibody 3F4 for humanized Tg mice. All animal experiments in this study were approved by the Institutional Animal Use and Care Committee and the Institutional Biosafety Committee.

### Preparation of brain homogenates

Frozen brain tissues from the frontal cortex of a patient with iatrogenic CJD (iCJD) and uninfected normal controls were obtained at autopsy. The characterization of this iCJD case has recently been reported^52^. Consent to use the autopsy brain tissue had been obtained in advance. The use of human brain tissues was authorized by the Institutional Review Board. Brain tissues from a mouse infected with mouse prion strain 139A and FVB wild-type mice were also used. A10% (w/v) infected human or mouse brain homogenate was prepared as described previously^21^,^53^, which was used as the PrP^Sc^ template for PMCA. To prepare the substrate for PMCA, brains from humanized transgenic mice described above were perfused with 5 mM EDTA in PBS and a 10% (w/v) brain homogenate was prepared as described^21^.

### Protein misfolding cyclic amplification

PMCA was performed as described with slight modifications^54^,^21^. In brief, the samples were subjected to PMCA, consisting of cycles of 30 min incubation at 37°C followed by a 40-second pulse of sonication at 60% potency for 18 h in a sonicator (QSONICA 700, Newtown, CT). To detect the amplified PrP^Sc^, 20 μl of PMCA-treated or untreated sample was incubated with 100 μg/ml PK for 70 min at 45°C. The reaction was terminated by adding PMSF to a final concentration of 5 mM and an equal amount of SDS sample buffer. Samples were then heated at 100°C for 10 min and a 10-μl sample was subjected to SDS-PAGE and Western blotting with 3F4 or 6D11.

### Scrapie-infected mouse neuroblastoma cell culture (ScN2a)

The effect of rHuPrP on PrP^Sc^ propagation in ScN2a cells was conducted as previously described with a minor modification^25^,^26^. Briefly, ScN2a cells seeded in six-well plates (5 × 10^5^ cells/well) containing 3 mL supplemented Opti-medium + 5%FBS were incubated with designated concentrations of recombinant proteins for 4 days. The cell lysates were prepared as previously described^34^. Samples of equal volumes containing equivalent amounts of protein were digested with 25 μg/mL proteinase K for 1 h at 37°C. Digestion was stopped by addition of PMSF to a final concentration of 2 μM and an equal volume of sample buffer was added. The samples were boiled for 10 min before loading onto 15% SDS-PAGE (SDS-polyacrylamide gel electrophoresis) precast Criterion gels (Bio-Rad, Hercules, CA, USA).

### Specific capture of abnormal PrP by g5p, recombinant PrP or anti-PrP antibodies

The preparation of g5p-, rPrP-, rPDI-, anti-PrP antibodies-magnetic beads and capture of PrP^C^ and PrP^Sc^ by the conjugated beads were performed as previously described^22^,^27^.

### Sodium dodecyl sulfate-polyacrylamide gel electrophoresis (SDS-PAGE) and immunoblotting

SDS-PAGE and immunoblotting was conducted as previously described^27^.

### Statistical analysis

Statistical significance of differences in PrP intensity was evaluated using Student's *t*-test. A difference was considered statistically significant if the *p* value was <0.05.

## Author Contributions

W.Q.Z. initiated and coordinated the entire project. J.Y., Y.A.Z., R.A., X.X., M.C.M., J.M., S.L., W.K.S., J.C., Q.K. and R.B.P. revised the manuscript. J.Y., Y.A.Z., R.A., Q.K., R.B.P., A.W. and W.Q.Z. conceived and designed the experiments. J.Y., Y.A.Z., R.A., X.X., X.Z., S.Z., Q.Z., R.B.P., A.W. and W.Q.Z. performed the experiments. J.Y., Y.A.Z., R.A., A.W., Q.K., R.B.P. and W.Q.Z. analyzed the data. G.K., J.M., S.L., W.K.S., J.C. and J.S. contributed reagents/materials/analysis tools. W.Q.Z. wrote the paper.

## Supplementary Material

Supplementary Information Supplementary Info

## References

[b1] Horiuchi M. & Caughey B. Prion protein interconversions and the transmissible spongiform encephalopathies. Structure 7, R231–R240 (1999). 10545332 10.1016/s0969-2126(00)80049-0

[b2] Prusiner S. B. *et al.* Transgenetic studies implicate interactions between homologous PrP isoforms in scrapie prion replication. Cell 63, 673–686 (1990). 1977523 10.1016/0092-8674(90)90134-z

[b3] Büeler H. *et al.* Normal development and behaviour of mice lacking the neuronal cell-surface PrP protein. Nature 356, 577–582 (1992). 1373228 10.1038/356577a0

[b4] Scott M. *et al.* Propagation of prions with artificial properties in transgenic mice expressing chimeric PrP genes. Cell 73, 979–988 (1993). 8098995 10.1016/0092-8674(93)90275-u

[b5] Scott M. R., Köhler R., Foster D. & Prusiner S. B. Chimeric prion protein expression in cultured cells and transgenic mice. Protein Sci. 1, 986–997 (1992). 1338978 10.1002/pro.5560010804 PMC2142161

[b6] Priola S. A., Caughey B., Race R. E. & Chesebro B. Heterologous PrP molecules interfere with accumulation of protease-resistant PrP in scrapie-infected murine neuroblastoma cells. J. Virol. 68, 4873–4878 (1994). 7913509 10.1128/jvi.68.8.4873-4878.1994 PMC236427

[b7] Bolton D. C. & Bendheim P. E. A modified host protein model of scrapie. Ciba. Found. Symp. 135, 164–181 (1988). 3136999 10.1002/9780470513613.ch11

[b8] Hope J. *et al.* The major polypeptide of scrapie-associated fibrils (SAF) has the same size, charge distribution and N-terminal protein sequence as predicted for the normal brain protein (PrP). EMBO J. 5, 2591–2597 (1986). 3096712 10.1002/j.1460-2075.1986.tb04539.x PMC1167157

[b9] Jarrett J. T. & Lansbury P. T. Jr. Seeding “one-dimensional crystallization” of amyloid: a pathogenic mechanism in Alzheimer's disease and scrapie? Cell 73, 1055–1058 (1993). 8513491 10.1016/0092-8674(93)90635-4

[b10] Caughey B. Scrapie associated PrP accumulation and its prevention: insights from cell culture. Br. Med. Bull. 49, 860–872 (1993). 8137133 10.1093/oxfordjournals.bmb.a072651

[b11] Telling G. C. *et al.* Prion propagation in mice expressing human and chimeric PrP transgenes implicates the interaction of cellular PrP with another protein. Cell 83, 79–90 (1995). 7553876 10.1016/0092-8674(95)90236-8

[b12] Kaneko K. *et al.* Evidence for protein X binding to a discontinuous epitope on the cellular prion protein during scrapie prion propagation. Proc. Natl. Acad. Sci. U S A 94, 10069–10074 (1997). 9294164 10.1073/pnas.94.19.10069 PMC23307

[b13] Tamgüney G. *et al.* Genes contributing to prion pathogenesis. J. Gen. Virol. 89, 1777–1788 (2008). 18559949 10.1099/vir.0.2008/001255-0 PMC2828448

[b14] Lee C. I., Yang Q., Perrier V. & Baskakov I. V. The dominant-negative effect of the Q218K variant of the prion protein does not require protein X. Protein Sci. 16, 2166–2173 (2007). 17766375 10.1110/ps.072954607 PMC2204135

[b15] Geoghegan J. C. *et al.* Trans-dominant inhibition of prion propagation in vitro is not mediated by an accessory cofactor. PLoS Pathog 5, e1000535 (2009). 19649330 10.1371/journal.ppat.1000535 PMC2713408

[b16] Xiao X. *et al.* Glycoform-selective prion formation in sporadic and familial forms of prion disease. PLoS One 8, e58786 (2013). 23527023 10.1371/journal.pone.0058786 PMC3602448

[b17] Nishina K. A. *et al.* The stoichiometry of host PrPC glycoforms modulates the efficiency of PrPSc formation in vitro. Biochemistry 45, 14129–14139 (2006). 17115708 10.1021/bi061526k

[b18] Bieschke J. *et al.* Autocatalytic self-propagation of misfolded prion protein. Proc. Natl. Acad. Sci. U S A 101, 12207–12211 (2004). 15297610 10.1073/pnas.0404650101 PMC514458

[b19] Kim J. I., Surewicz K., Gambetti P. & Surewicz W. K. The role of glycophosphatidylinositol anchor in the amplification of the scrapie isoform of prion protein in vitro. FEBS Lett. 583, 3671–3675 (2009). 19854187 10.1016/j.febslet.2009.10.049 PMC2856614

[b20] Watts J. C. *et al.* Interactome analyses identify ties of PrP and its mammalian paralogs to oligomannosidic N-glycans and endoplasmic reticulum-derived chaperones. PLoS Pathog. 5, e1000608 (2009). 19798432 10.1371/journal.ppat.1000608 PMC2749441

[b21] Zhou X. *et al.* Alkylating antitumor drug mechlorethamine conceals a structured PrP domain and inhibits in vitro prion amplification. J. Toxicol. Environ. Health A. 74, 1493–1503 (2011). 22043910 10.1080/15287394.2011.618978

[b22] Zou W. Q. *et al.* Antibody to DNA detects scrapie but not normal prion protein. Proc. Natl. Acad. Sci. U S A 101, 1380–1385 (2004). 14734804 10.1073/pnas.0307825100 PMC337061

[b23] Morillas M., Swietnicki W., Gambetti P. & Surewicz W. K. Membrane environment alters the conformational structure of the recombinant human prion protein. J. Biol. Chem. 274, 36859–36865 (1999). 10601237 10.1074/jbc.274.52.36859

[b24] Caughey B. *et al.* Prion protein biosynthesis in scrapie-infected and uninfected neuroblastoma cells. J. Virol. 63, 175–181 (1989). 2562814 10.1128/jvi.63.1.175-181.1989 PMC247670

[b25] Doh-Ura K., Iwaki T. & Caughey B. Lysosomotropic agents and cysteine protease inhibitors inhibit scrapie-associated prion protein accumulation. J Virol. 74, 4894–4897 (2000). 10775631 10.1128/jvi.74.10.4894-4897.2000 PMC112015

[b26] Fernaeus S., Reis K., Bedecs K. & Land T. Increased susceptibility to oxidative stress in scrapie-infected neuroblastoma cells is associated with intracellular iron status. Neurosci Lett. 389, 133–136 (2005). 16095817 10.1016/j.neulet.2005.07.032

[b27] Yuan J. *et al.* Insoluble aggregates and protease-resistant conformers of prion protein in uninfected human brains. J. Biol. Chem. 281, 34848–34858 (2006). 16987816 10.1074/jbc.M602238200

[b28] Horiuchi M., Priola S. A., Chabry J. & Caughey B. Interactions between heterologous forms of prion protein: binding, inhibition of conversion, and species barriers. Proc. Natl. Acad. Sci. U S A 97, 5836–5841 (2000). 10811921 10.1073/pnas.110523897 PMC18520

[b29] Haraguchi T. *et al.* Asparagine-linked glycosylation of the scrapie and cellular prion proteins. Arch Biochem Biophys. 274, 1–13 (1989). 2505674 10.1016/0003-9861(89)90409-8

[b30] Stahl N., Borchelt D. R., Hsiao K. & Prusiner S. B. Scrapie prion protein contains a phosphatidylinositol glycolipid. Cell 51, 229–240 (1987). 2444340 10.1016/0092-8674(87)90150-4

[b31] Priola S. A. & Lawson V. A. Glycosylation influences cross-species formation of protease-resistant prion protein. EMBO J. 20, 6692–6699 (2001). 11726505 10.1093/emboj/20.23.6692 PMC125748

[b32] Priola S. A. Species barriers in prion disease. Prions and Diseases. Zou W. Q., & Gambetti P. (eds.), 139–154 (Springer Science, New York, 2013).

[b33] Chasseigneaux S. *et al.* V180I mutation of the prion protein gene associated with atypical PrPSc glycosylation. Neurosci. Lett. 408, 165–169 (2006). 17029785 10.1016/j.neulet.2006.08.008

[b34] Zou R. S. *et al.* Characterization of spontaneously generated prion-like conformers in cultured cells. Aging 3, 968–984 (2011). 21990137 10.18632/aging.100370 PMC3229973

[b35] Atarashi R. *et al.* Ultrasensitive detection of scrapie prion protein using seeded conversion of recombinant prion protein. Nat Methods. 4, 645–650 (2007). 17643109 10.1038/nmeth1066

[b36] Atarashi R. *et al.* Simplified ultrasensitive prion detection by recombinant PrP conversion with shaking. Nat Methods. 5, 211–212 (2008). 18309304 10.1038/nmeth0308-211

[b37] Kim J. I. *et al.* Mammalian prions generated from bacterially expressed prion protein in the absence of any mammalian cofactors. J Biol Chem. 285, 14083–14087 (2010). 20304915 10.1074/jbc.C110.113464 PMC2863186

[b38] Wang F., Wang X., Yuan C. G. & Ma J. Generating a prion with bacterially expressed recombinant prion protein. Science 327, 1132–5 (2010). 20110469 10.1126/science.1183748 PMC2893558

[b39] Deleault N. R. *et al.* Isolation of phosphatidylethanolamine as a solitary cofactor for prion formation in the absence of nucleic acids. Proc Natl Acad Sci U S A. 109, 8546–8551 (2012). 22586108 10.1073/pnas.1204498109 PMC3365173

[b40] Makarava N. *et al.* Recombinant prion protein induces a new transmissible prion disease in wild-type animals. Acta Neuropathol. 119, 177–187 (2010). 20052481 10.1007/s00401-009-0633-x PMC2808531

[b41] Kocisko D. A. *et al.* Cell-free formation of protease-resistant prion protein. Nature 370, 471–474 (1994). 7913989 10.1038/370471a0

[b42] Caughey B. Prion protein interconversions. Philos. Trans. R. Soc. Lond. B Biol. Sci. 356, 197–202 (2001). 11260800 10.1098/rstb.2000.0765 PMC1088425

[b43] Baron G. S. *et al.* Conversion of raft associated prion protein to the protease-resistant state requires insertion of PrP-res (PrP(Sc)) into contiguous membranes. EMBO J. 21, 1031–1040 (2002). 11867531 10.1093/emboj/21.5.1031 PMC125906

[b44] Kirby L. *et al.* In vitro cell-free conversion of bacterial recombinant PrP to PrPres as a model for conversion. J. Gen. Virol. 84, 1013–1020 (2003). 12655105 10.1099/vir.0.18903-0

[b45] Chesebro B. *et al.* Anchorless prion protein results in infectious amyloid disease without clinical scrapie. Science 308, 1435–1439 (2005). 15933194 10.1126/science.1110837

[b46] Korth C. *et al.* Prion (PrPSc)-specific epitope defined by a monoclonal antibody. Nature 390, 74–77 (1997). 9363892 10.1038/36337

[b47] Zou W. Q. & Cashman N. R. Acidic pH and detergents enhance in vitro conversion of human brain PrPC to a PrPSc-like form. J. Biol. Chem. 277, 43942–43947 (2002). 12161431 10.1074/jbc.M203611200

[b48] Furuya K. *et al.* Intracerebroventricular delivery of dominant negative prion protein in a mouse model of iatrogenic Creutzfeldt-Jakob disease after dura graft transplantation. Neurosci. Lett. 402, 222–226 (2006). 16759805 10.1016/j.neulet.2006.03.062

[b49] Abskharon R. N. *et al.* A novel expression system for production of soluble prion proteins in E. coli. Microb. Cell Fact. 11, 6 (2012). 22233534 10.1186/1475-2859-11-6 PMC3283519

[b50] Rancy P. C. & Thorpe C. Oxidative protein folding in vitro: a study of the cooperation between quiescin-sulfhydryl oxidase and protein disulfide isomerase. Biochemistry 47, 12047–12056 (2008). 18937500 10.1021/bi801604x PMC2892342

[b51] Fischer M. *et al.* Prion protein (PrP) with amino-proximal deletions restoring susceptibility of PrP knockout mice to scrapie. EMBO J. 15, 1255–1264 (1996). 8635458 PMC450028

[b52] Mikol J. *et al.* Creutzfeldt-Jakob disease with unusually extensive neuropathology in a child treated by native human growth hormone. Clin. Neuropath. 31, 127–134 (2012). 10.5414/NP300441 PMC3693083 22551916

[b53] Nishida N. *et al.* Successful transmission of three mouse-adapted scrapie strains to murine neuroblastoma cell lines overexpressing wild-type mouse prion protein. J Virol. 74, 320–325 (2000). 10590120 10.1128/jvi.74.1.320-325.2000 PMC111542

[b54] Castilla J., Saá P., Hetz C. & Soto C. In vitro generation of infectious scrapie prions. Cell 121, 195–206 (2005). 15851027 10.1016/j.cell.2005.02.011

