# Supplementary material for: Recombinant Human Prion Protein Inhibits Prion Propagation in vitro
Source: Sci Rep. 2013 Oct 9;3:2911. doi: 10.1038/srep02911 (PMC3793212; doi:10.1038/srep02911)
Supplement: Supplementary Information — Supplementary Info [file srep02911-s1.pdf]

Recombinant Human Prion Protein Inhibits Prion Propagation *in vitro*

Jue Yuan[1,3*], Yi-An Zhan[1,7*], Romany Abskharon[5,6,14*], Xiangzhu Xiao[1,3], Manuel Camacho Martinez[1,3], Xiaochen Zhou[1,7], Geoff Kneale[8], Jacqueline Mikol[9], Sylvain Lehmann[10], Witold K. Surewicz[13], Joaquín Castilla[12], Jan Steyaert[5,6], Shulin Zhang[1], Qingzhong Kong[1,2,3], Robert B. Petersen[1,2,11], Alexandre Wohlkonig[5,6**] & Wen-Quan Zou[1**,2,3,4,7]

Departments of [1]Pathology, [2]Neurology, [11]Neuroscience, [13]Physiology and Biophysics, [3]National Prion Disease Pathology Surveillance Center, [4]National Center for Regenerative Medicine, Case Western Reserve University School of Medicine, Cleveland, Ohio, USA

[5]VIB, Department of Structural Biology, [6]Structural Biology Brussels, Vrije Universiteit Brussels, Belgium

[7]The First Affiliated Hospital, Nanchang University, Nanchang, Jiangxi Province, The People's Republic of China

[8]Biophysics Laboratories, Institute of Biomedical and Biomolecular Sciences, University of Portsmouth, Portsmouth, United Kingdom

[9]Hôpital Lariboisière, Service d'Anatomie et Cytologie Pathologiques, Paris Denis Diderot University, Paris, France

[10]IRB - Hôpital ST ELOI, CHU de Montpellier, Montpellier, France

[12]CIC bioGUNE and IKERBASQUE, Basque Foundation for Science, 48160 Derio and 48011 Bilbao, Bizkaia, Spain

[14]National Institute of Oceanography and Fisheries (NIFO), Cairo, Egypt

Correspondence and requests for materials should be addressed to W.Q.Z. (wenquan.zou@case.edu) or A.W. (awohlkon@vub.ac.be)

Running title: Recombinant PrP and prion amplification

*These authors contributed equally to this work.

Figure legend

Figure S1│Effect of rMoPrP23-231 or rHuPrP23-231 on amplification of mouse PrP^{Sc} in PMCA. PMCA was performed with mouse prion 139A as seeds and brain homogenates from wild-type FVB mouse as substrates in the presence of different concentrations of rMoPrP23-231 or rHuPrP23-231. (A) Western blotting of PK-resistant PrP^{Sc} with the 6D11 antibody after PMCA.

(B) Densitometric analysis of PK-resistant PrP^{Sc} after PMCA in the presence of different amounts of rMoPrP or rHuPrP based on three independent experiments. While rMoPrP23-231 significantly inhibited mouse PrP^{Sc} amplification, rHuPrP23-231 exhibited no significant inhibition (*: $p < 0.05$; **: $p < 0.01$).

Figure S2│Binding of various recombinant PrP molecules to human PrP^{Sc}. Capture of human PrP^{Sc} from CJD brain homogenate was performed by incubation of various magnetic beads conjugated with rHuPrP23-231, rHuPrP90-231, rHuPrP23-145, or rMoPrP23-231. G5p beads were used as a control. (A) Western blotting of PrP^{Sc} captured by recombinant PrP. The captured PrP was treated with or without PK at 50 µg/ml and the blot was probed with 3F4. All recombinant PrP molecules captured human PrP^{Sc}, while rHuPrP23-145 captured highest amount of PrP^{Sc} compared to other recombinant PrP species. (B) Short exposure of Western blot of PrP^{Sc} captured by rHuPrP23-145 shown in panel (A) to reveal the typical three PK-resistant PrP fragments. The blot is a representative of two independent experiments.

Figures

Figure S1

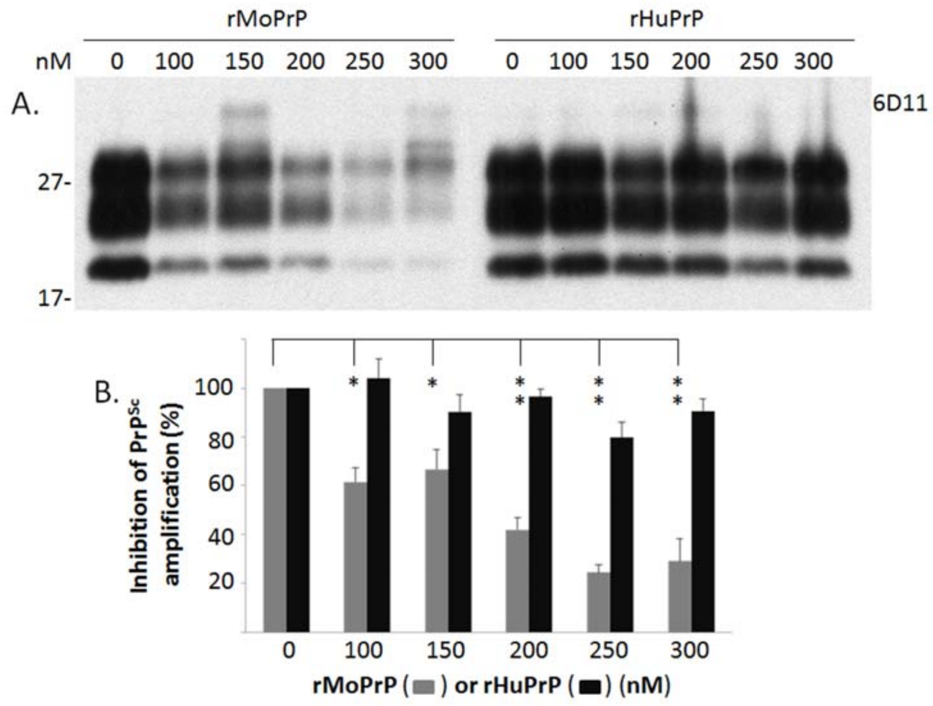

Figure S2

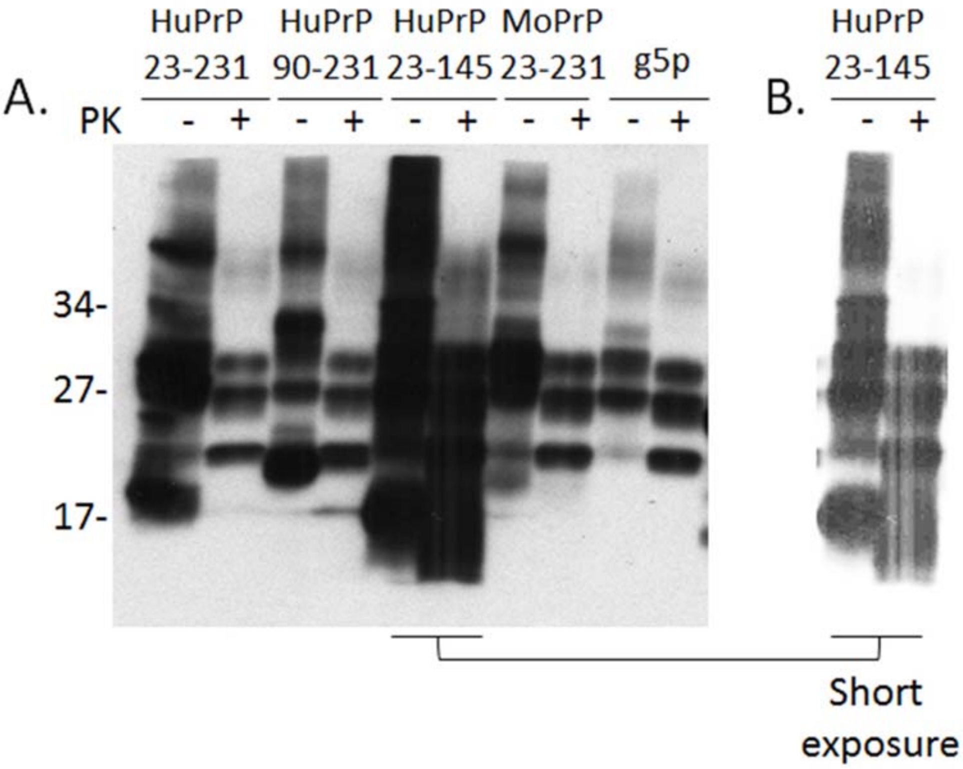
HuPrP 23-231
HuPrP 90-231
HuPrP 23-145
MoPrP 23-231
g5p
A.
PK
- + - + - + - + - +
34-
27-
17-
B.
HuPrP 23-145
- +
Short exposure
